# A semi-automated luminescence based standard membrane feeding assay identifies novel small molecules that inhibit transmission of malaria parasites by mosquitoes

**DOI:** 10.1038/srep18704

**Published:** 2015-12-21

**Authors:** Martijn W. Vos, Will J. R. Stone, Karin M. Koolen, Geert-Jan van Gemert, Ben van Schaijk, Didier Leroy, Robert W. Sauerwein, Teun Bousema, Koen J. Dechering

**Affiliations:** 1 TropIQ Health Sciences, PO Box 9101, 6500 HB Nijmegen, The Netherlands; 2 Department of Medical Microbiology, Radboud University Nijmegen Medical Center, 6500 HB Nijmegen, The Netherlands; 3 Medicines for Malaria Venture, 20 Route de Pré-Bois, 1215 Geneva 15, Switzerland

## Abstract

Current first-line treatments for uncomplicated *falciparum* malaria rapidly clear the asexual stages of the parasite, but do not fully prevent parasite transmission by mosquitoes. The standard membrane feeding assay (SMFA) is the biological gold standard assessment of transmission reducing activity (TRA), but its throughput is limited by the need to determine mosquito infection status by dissection and microscopy. Here we present a novel dissection-free luminescence based SMFA format using a transgenic *Plasmodium falciparum* reporter parasite without resistance to known antimalarials and therefore unrestricted in its utility in compound screening. Analyses of sixty-five compounds from the Medicines for Malaria Venture validation and malaria boxes identified 37 compounds with high levels of TRA (>80%); different assay modes allowed discrimination between gametocytocidal and downstream modes of action. Comparison of SMFA data to published assay formats for predicting parasite infectivity indicated that individual *in vitro* screens show substantial numbers of false negatives. These results highlight the importance of the SMFA in the screening pipeline for transmission reducing compounds and present a rapid and objective method. In addition we present sixteen diverse chemical scaffolds from the malaria box that may serve as a starting point for further discovery and development of malaria transmission blocking drugs.

The introduction of Artemisinin combination therapy (ACT) as the first line treatment for malaria has contributed substantially to recent declines in child mortality across Africa^1^,^2^. ACT rapidly clear the *Plasmodium* life stages responsible for clinical disease but at the dose used to clear asexual blood stage parasites have only limited effect against mature gametocytes, the stage transmitted to mosquitoes^3^. Though immature gametocytes may be cleared shortly after their production^4^ resulting in reduced gametocyte burden and transmission potential^5^, current dosing regimens of ACT do not radically clear mature transmission stages and do not prevent transmission shortly after treatment^6^. Expanding efforts to achieve local malaria elimination and contain transmission in areas of emerging ACT resistance have stimulated interest in combining standard treatment with compounds active against mature gametocytes^7^ and have highlighted the need to screen new antimalarial drug candidates for their transmission reducing activity (TRA).

Currently the only available drug that radically prevents malaria transmission is primaquine^8^, but its wide-scale deployment is hampered by issues of safety and dosing^9^,^10^. Though the results of numerous ongoing trials give reason to expect wider scale use of single-low dose primaquine for reducing *P. falciparum* transmission, there is growing interest in identifying novel transmission reducing compounds^7^.

For screening new compounds, several assays determining gametocyte viability activity have been developed. These have been based on the expression of metabolic markers or reporter genes, or more recently on gametocyte activation *in vitro* measured by the presence of female and male gametes^11^,^12^,^13^. Such assays are, however, not always predictive of blocking transmission of the parasite to the mosquito, and agents with putative TRA are best tested in the standard membrane feeding assay (SMFA). In the SMFA cultured gametocytes are mixed with blood and test compounds, and this mix is fed to laboratory reared mosquitoes to determine the effect of the compound on the parasites establishment in the mosquito. As the outcome of the SMFA is mosquito infection and the timing and duration of compound exposure is controlled, the assay is easily adapted to assess effects active at any stage of parasite development – from gametocyte inhibition, to inhibition of sporogony^13^,^14^. The SMFA has numerous pitfalls though, first among these being the need to dissect mosquitoes and count oocysts on the mosquito midgut by microscopy; a labour intensive and subjective task, which imposes significant limits on the assays scalability. We previously described the use of a parasite line expressing a fusion of the green fluorescent and firefly luciferase proteins (NF54HT-GFP-Luc) in a new SMFA format based on the measurement of luciferase activity in groups of homogenised individual or pooled mosquitoes^15^. Although very effective, this strain may be sub-optimal for drug screening; introduction of the human dihydrofolate reductase (hDHFR) selection marker during integration of the GFP-Luc expression cassette renders it resistant to antifolates^16^ and makes it necessary to maintain exposure to antifolate compound WR99210 during parasite culture to prevent reversion to wild-type (WT), possibly modifying its response to other inhibitors by affecting parasite metabolism. Here we report the development of a parasite line that constitutively expresses GFP-Luc and is free of limiting selection markers, for use in an efficient, dissection-free SMFA screen. Our approach used frozen mosquitoes and machine homogenisation in a 96-well format to improve the efficiency of indiscriminate drug screening. After validation and comparison of luciferase expression with microscopy based oocyst counts we used our luminescent SMFA to screen 47 marketed and experimental antimalarials and compared the results with data from the gametocytocidal lactate dehydrogenase (pLDH) activity assay and Pfs25 AlphaLISA assay. Furthermore, the luciferase screen was used to evaluate the TRA of 18 compounds selected from the Medicines for Malaria Venture (MMV) malaria box specifically for their potential activity as gametocytocidals. In this final screen, gametocytocidal or permanent inhibitory activity was confirmed by incubating gametocytes with compounds before ‘washing out’ the compound prior to mosquito feeding, while specific activity against the parasite stages emerging in the mosquito gut was confirmed by adding compounds to the gametocyte culture immediately prior to mosquito feeding. These assays identified novel transmission blocking small molecules and provide new insight into the predictive value of *in vitro* metabolic and gamete formation inhibition assays, showing they can miss potent compounds, and highlighting the advantages of the gold-standard SMFA for screening transmission blocking compounds. Importantly, our more scalable luciferase based read-out increases throughput of the assay and makes compound screening with the SMFA less resource intensive than when performed with microscopy.

## Results

### Generation of a GFP-Luc expressing parasite line free of selection markers

**Figure 1:**
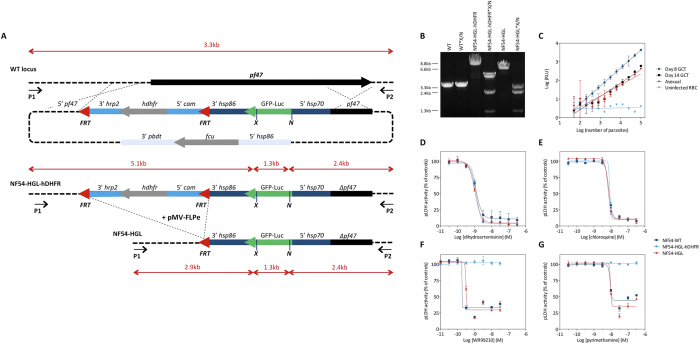
(**A**) Schematic of the gene targeting strategy to insert a GFP-Luc reporter gene under control of the *hsp70* promoter in the *pfs47* locus, showing the genomic locus of the wild-type (WT) and gene deletion mutants ∆pf47 containing the GFP-luciferase ORF before and after removal of the *hdhfr* resistance marker. *P1, P2*: primers MWV300 and MWV301, respectively, used for PCR analysis; *X* (*XcmI*), *N* (*NcoI*): restriction sites used for conformation of LR-PCR analysis; *pf47*: *pf47* locus; *hrp:* histidine rich protein; *cam:* calmodulin; *hsp:* heat shock protein; *hdhfr*: human dihydrodrofolate reductase coding region; *GFP-Luc*: GFP-luciferase fusion protein; *fcu:* cytosine deaminase/uracil phosphoribosyl transferase; *pbdt: P. berghei dhfr* transcription termination region; kb: kilo basepairs. (**B**) Verification of the integration site by PCR and restriction digestion. Genomic DNA fragments from the parental NF54 strain (WT), intermediate transgene NF54-HGL-hDHFR and final transgene NF54-HGL were amplified by PCR using primers MWV300 and MWV301 (P1 and P2) and analysed by DNA gel electrophoresis. Where indicated, the PCR fragment was subjected to restriction digestion with *XcmI/NcoI* (X/N) (**C**) Luminescence intensity (relative light units, RLU) as a function of cell number. The figure shows a comparison between uninfected red blood cells (RBC), immature gametocytes (Day 8 GCT), mature gametocytes (Day 14 GCT) and asexual blood stage parasites. (**D–G**) FLP-FRT mediated excision of the selection marker restores sensitivity to antifolates. Drug sensitivity analysis of WT, ∆Pf47GFP-luc-hdhfr and ∆Pf47GFP-luc to DHA (**D**), Chloroquine (**E**), Pyrimethamine (**F**), and WR99210 (**G**) in a dose dependent asexual growth inhibition assay using pLDH enzyme activity as a readout. Depicted data represents a triplicate measurement and was normalized to the MIN (10 μM DHA) and MAX (0.1% DMSO) controls. Error bars indicate standard deviations.

To allow unrestrained evaluation of new compounds we developed a new parasite line, free of drug selection markers (full title: NF54-∆Pf47-5′*hsp70*-GFP-Luc; hereafter: NF54-HGL), that stably expresses a GFP-Luciferase fusion protein under control of the constitutive *P. falciparum hsp70* promoter (Fig. 1A,B).

To confirm the proposed effect of removal of the hDHFR selection marker on antifolate sensitivity, we compared sensitivity of wildtype NF54, intermediate clone NF54-HGL-hDHFR (created prior to FLP recombinase-mediated excision of the hDHFR maker) and NF54-HGL asexual blood stage parasites to dihydroartemisinin (DHA), chloroquine, and two antifolate drugs: WR99210 and Pyrimethamine. The half maximal inhibitory concentration (IC50) for DHA and chloroquine are comparable for all three parasite lines. As predicted, presence of the hDHFR selection marker in NF54-HGL-hDHFR renders this parasite resistant to WR99210 and Pyrimethamine. Excision of the hDHFR open reading frame effectively restores sensitivity to these antifolates for NF54-HGL (Fig. 1D–G). Since pLDH is a stable enzyme and antifolates have relatively slow parasite killing kinetics, the inhibition by WR99210 and pyrimethamine did not reach 100% within the timeframe of the assay^17^.

### Assessment of GFP-Luc expression

Analyses of luminescence signals confirmed expression of the GFP-Luc protein in asexual blood stage parasites (Fig. 1C). In line with previous reports on different expression levels in the various life cycle stages of the parasite^18^, expression was higher in day 8 gametocytes than in mature gametocytes and asexual blood stages. To assess GFP-Luc expression during the parasites development in mosquitoes, *Anopheles stephensi* were fed a blood-meal containing NF54-HGL gametocytes and dissected at various time-points post infection (PI) to allow visual inspection of the mosquito midgut. No fluorescence was observed during ookinete formation (24 h PI, data not shown), indicating that GFP expression levels may have been under the limit for microscopical detection. From day 6 PI GFP positive oocysts were easily observable (Supplementary Fig. S1). Luciferase expression was weak during early sporogonic development (2–6 days PI, data not shown), but robust signal in low intensity infections (mean oocysts = 1.28, range = 0–7) was detectable from day 7 PI onwards (Supplementary Fig. S2). Uninfected negative control mosquitoes showed low levels of luminescence (n = 617; mean RLU = 3.2 RLU; standard deviation = 1.9 RLU). Day 8 PI showed a clear separation of positive and negative mosquitoes and was chosen for subsequent assessments of infection intensity (*i.e.* number of oocysts per midgut) and prevalence (*i.e.* percentage of mosquitoes with oocyst stage infection) based on luminescence intensity.

### Microscopy and luciferase based assessments of infection and TRA

To confirm that measurements of luciferase activity can replace manual dissection-based oocyst counts, as was previously shown for NF54HT-GFP-Luc^15^, 172 experimental mosquito feeds (hereafter ‘feeds’) were performed using NF54-HGL gametocyte culture. From each feed, separate mosquito samples were assayed by standard microscopy and by luminescent SMFA (microscopy, n = 2557; luminescence, n = 3570). Thirty-two of these feeds were performed as controls allowing for direct comparison of infection intensity and prevalence based on oocyst and luminescence readouts. These were performed either without test compounds (0.1% DMSO, n = 16) or with control compound DHA that shows gametocytocidal activity at high concentration (10 μM DHA, n = 16)^13^. Mean oocyst counts for control feeds were between zero and 29 oocysts (mean 4.9 oocysts), and had a positive linear relationship with mean luminescence intensity (R^2^ = 0.86, p < 0.0001). Similarly, oocyst prevalence was positively associated with luminescence based prevalence (R^2^ = 0.95, p < 0.0001) (Supplementary Fig. S3). Combining all mosquitoes from control feeds, prevalence estimates for all mosquitoes from control feeds were 43.33% (208/480) for microscopy, and 49.22% (350/711) for luminescence.

**Figure 2:**
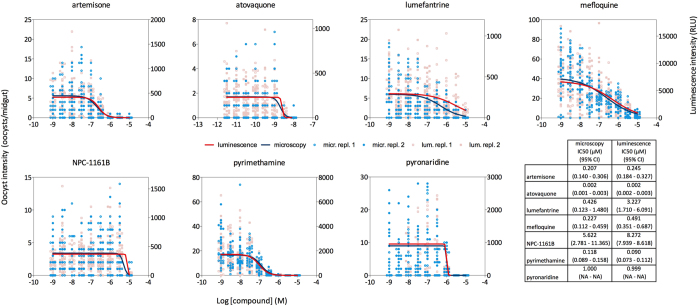
Standard Membrane Feeding Experiments for 7 compounds from the MMV validation box, assessed both by microscopy and luciferase assay. All compounds were tested in nine dilutions in duplicate. The figure shows oocyst counts in individual mosquitoes and relative light units (RLU) from a distinct cohort of mosquitoes from the same cage. The table shows IC50 values and cognate 95% confidence intervals (CI) determined from fitting the data to a logistic regression model by Maximum Likelihood Estimation. NA: confidence interval not computed as the model did not converge.

To further validate the luminescent SMFA we tested seven compounds from the MMV validation box in full dose response in an ‘indirect’ luminescent SMFA format. In the indirect assay, gametocytes were incubated with compounds for 24 hours, and mosquitoes were fed a blood-meal containing this gametocyte culture/compound mix. Microscopic examination of oocyst densities in midguts and luminescence measurements were performed on distinct cohorts of mosquitoes from the same cage. As shown in Fig. 2, all compounds dose-dependently reduced oocyst formation and luminescence intensity. Data were fitted to a Hill equation using Maximum Likelihood Estimation to find the best fit. The dose response curves fitted on the microscopy and luminescence data aligned very well for artemisone, atovaquone, mefloquine, NPC1161B, pyrimethamine and pyronaridine. The curves for lumefantrine appeared to be less well aligned due to a distinct dispersion of oocyst counts and RLU values at the highest concentration tested. For all other compounds the derived IC50 values differ by no more than 2–3 fold when comparing the two read-outs.

### The luminescent SMFA identifies compounds with different modes of TRA

**Figure 3:**
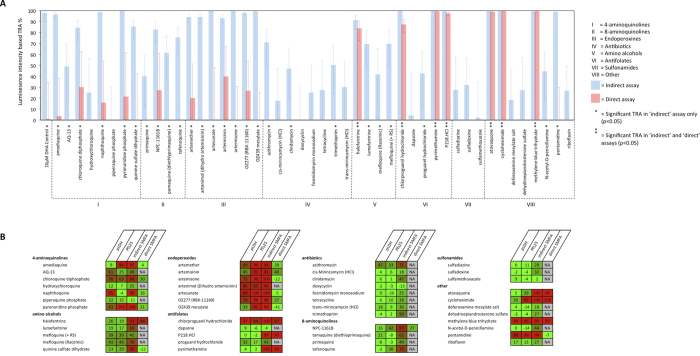
(**A**) Single concentration (5 μM) rapid luminescent SMFA screen of 47 compound from MMV validation box, assessed with the indirect and direct assay variants. TRA% was calculated from relative differences between test and control feed luminescence intensity. Error bars indicate 95% confidence intervals. (**B**) Heat map comparing the SMFA data to published data from pLDH and Pfs25-based gametocyte viability assays. The numbers indicate percentage inhibition at a compound concentration of 5 μM. A color scale from green to red indicates low to high inhibition.

Using only luminescence readouts, we screened 47 established antimalarial compounds in the indirect luminescent SMFA format at a 5 μM concentration. Samples of 22–24 mosquitoes were analysed for each compound (n = 46*24; 1*22). The results of this rapid screen are shown in Fig. 3A, with TRA (%) calculated using generalised linear mixed models (GLMM) from differences in the intensity of infection between test and vehicle (0.1% DMSO) control feeds. Thirty-four compounds had statistically significant TRA based on reductions in luminescence intensity; 21/34 with ≥ 80% TRA^15^.

To provide more information on the mechanism of active compounds, all compounds showing ≥ 80% TRA on the intensity scale in the indirect assay were re-tested, this time adding compounds to the blood/gametocyte culture mix immediately prior to mosquito feeding (hereafter, the ‘direct’ assay variant). Fourteen compounds had > 80% TRA in the indirect assay but not in the direct assay, suggesting the absence of strong activity against the parasite life stages occurring in the mosquito gut. Amino alcohol halofantrine, antifolates P218, pyrimethamine and chlorproguanil, synthetic dye methylene blue, BC1 inhibitor atovaquone, and protein synthesis inhibitor cycloheximide had comparable TRA in the indirect and direct SMFA formats, indicating activity against mosquito stage parasites, but not excluding the possibility of concurrent gametocytocidal action.

### The luminescent SMFA identifies compounds with TRA that are missed by gametocyte and gametogenesis assays

The results of the indirect and direct assays were compared with previously described data^13^ showing the effect of the same 47 compounds at 5 μM in a gametocyte viability assay with pLDH readout, and a homogeneous immunoassay detecting the Pfs25 antigen on emerging female gametes (Fig. 3B). At a threshold of ≥80% inhibition, the pLDH and gametogenesis assays identified 6/47 and 13/47 compounds respectively as having gametocytocidal activity. The activity of all but one of the six compounds identified by the pLDH assay (napthoquine) was confirmed by the gametogenesis assay, while all hits from either viability assay were confirmed in the indirect luminescent SMFA. However, high TRA was observed for a number of compounds where the inhibition assays showed low activity, indicating that some compounds had activity only identifiable with transmission as the definitive readout. Total false negative rates for the pLDH and gametogenesis assays were 32% (15/47) and 17% (8/47), respectively. Excluding compounds that blocked transmission in the direct luminescent SMFA, which we presumed to have activity downstream of the gametocyte, the SMFA still identified a larger number of transmission-blocking compounds than either gametocytocidal assay, with false negativity rates of 25% (10/40) in the pLDH assay and 10% (4/40) in the gametogenesis assay. This is not surprising since the SMFA covesr a higher biological content than either of the other two methodologies.

### Identification of novel transmission-blocking small molecules

**Figure 4:**
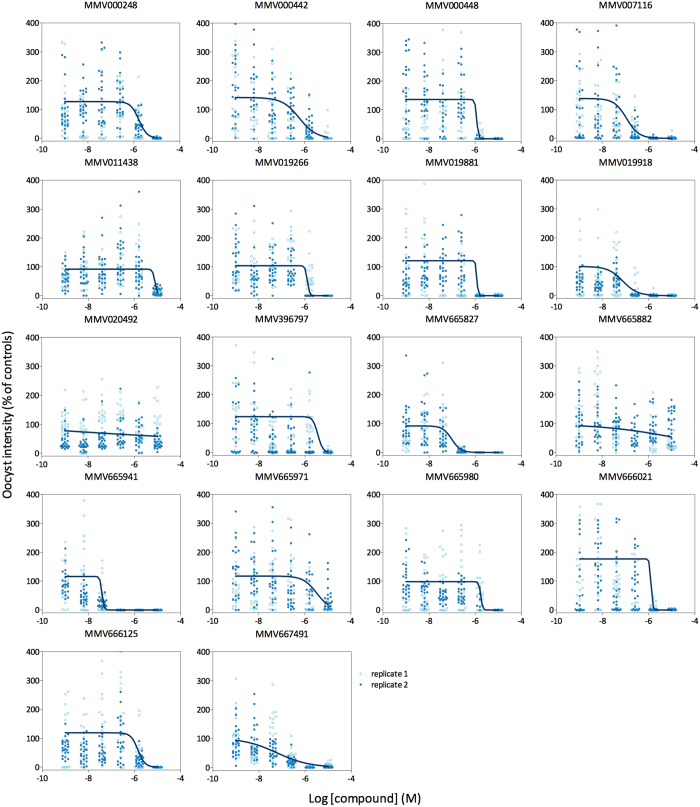
Dose response experiments for 18 compounds from the MMV malaria box, assessed in the luminescent SMFA in a wash-out assay variant. All compounds were tested in duplicate in two independent experiments. The figure shows oocyst intensities relative to vehicle control (0.1% DMSO) as a function of concentration for the compounds indicated at the top of the panels.

In recent years, a number of gametocyte assays have been developed that attempt to predict gametocyte viability and infectivity based on metabolic activity, reporter gene expression, or changes in cell shape or antigen expression^11^,^12^,^13^,^19^,^20^,^21^,^22^,^23^,^24^,^25^,^26^. In an effort coordinated by the Bill & Melinda Gates Foundation and the Medicines for Malaria Venture (MMV), a number of these and additional novel assays selected a subset of compounds from the MMV Malaria box as potentially transmission blocking. All of these molecules kill asexual blood stage parasites with submicromolar IC50s (Table 1). Here, we evaluated 18 of these selected compounds in full dose-response experiments using the luminescent SMFA. In order to confirm a gametocyte-based mechanism of action, compounds were washed out following incubation with stage V gametocytes to prevent activity against parasite stages emerging in the mosquito midgut. Effectiveness of the washing step was tested by evaluating the activity of pyrimethamine, which has potent activity against the parasite stages that develop in the mosquito midgut (Fig. 2) but not against gametocytes^27^. Indeed, washing a culture of gametocytes exposed to pyrimethamine restored subsequent oocyst development in the mosquito, confirming that this format of the assay is likely restricted to the analyses of gametocytocidal modes of actions (Supplementary Fig. S4). This might however be different for more lipophilic drugs, which might be washed out with less efficiency. Sixteen out of the 18 Malaria box compounds tested in this wash-out mode of the SMFA dose-dependently reduced oocyst development in the mosquito midgut (Fig. 4). Compounds MMV665941, MMV667491, MMV019918 and MMV665827 were among the most potent transmission-reducing molecules, with IC50 values of 0.04, 0.06, 0.07 and 0.1 μM, respectively. To reveal whether these compounds act against the parasite stages that develop in the mosquito midgut on top of their gametocytocidal activity, all compounds were tested in the direct assay variant at the same maximum concentration as in the wash-out assay (10 μM), confirming that 10/18 compounds significantly reduced mosquito oocyst intensity Compounds MMV020492 and MMV665882 that did not show gametocytocidal activity were equally inactive in the direct SMFA. To further address the mechanism of action of compounds with functional TRA, the SMFA data were compared to data from published studies that addressed different aspects of gametocyte biology (Table 1). MMV019918 was the only compound consistently showing activity across the different assay formats, although IC50 values ranged from 0.07 μM to 0.9 μM, with large differences between the two assays that interrogated the ability of a gametocyte to undergo exflagellation. Conversely, MMV007116 and MMV665827 showed potent activity in the SMFA but very low activity in the published gametocyte assays, with the exception of a luciferase reporter assay that revealed the effect of these compounds on gametocyte viability. In general, the luminescent SMFA identified more compounds with functional TRA than predicted by each of the individual published gametocyte assay formats.

## Discussion

The firefly luciferase expressing parasite line NF54-HGL enables the SMFA to be performed for indiscriminate compound screening with a semi-automated luciferase based read-out. The results of the current study demonstrate the versatility of the SMFA for screening compounds with activity against different transmission stages of the malaria parasite, and validate the luminescent assay format in combination with microtiter plate-based processing of mosquitoes for improving the efficiency of the biological gold-standard for assessing *Plasmodium* transmission in controlled experiments.

We presented a screening pipeline for the SMFA that allowed us to investigate drug mechanism of action while retaining transmission as the assay end-point. The indirect assay, in which compounds were incubated with gametocytes for 24 hours prior to mosquito feeding, represents the simplest and most sensitive screen and the first step in the pipeline for screening large compound libraries. The direct assay, in which the compound is added at the moment the gametocyte activates to form a gamete, interrogates compound action in the mosquito midgut. Pharmacokinetics in the mosquito are largely unknown, but detoxifying enzymes may limit the compound’s action in the midgut, restricting the direct SMFA to monitoring effects on gametogenesis, ookinete formation and perhaps early sporogony^28^. Lastly, the wash-out format of the SMFA addresses a mode of action that is likely restricted to the gametocyte. High-throughput screening is impossible with the SMFA, but the luminescent SMFA represents a significant improvement in the techniques scalability. The replacement of direct oocyst counts with luciferase measurements from homogenised mosquitoes in the SMFA was described previously^15^ for parasite line NF54HT-GFP-luc^16^, but aspects of the methodology described here differ. The current reporter parasite is devoid of the DHFR selection marker, allowing for precise description of the effects of antifolates including pyrimethamine on oocyst development, and expediting the evaluation of a wide variety of compound libraries. Mosquito sample sizes were limited to increase throughput, and freezing mosquitoes prior to further processing increased flexibility of laboratory operations. Furthermore, semi-automated processing of mosquitoes significantly reduced the time needed for assessment of infection status. In all, the luminescent format for individual mosquitoes reduces hands-on time 2-fold in comparison with the microscopical readout, which is a tremendous step up from a previously published ‘industrialized’ SMFA format^29^. For even further increased throughput, screening in the luminescent SMFA may be performed by pooling larger numbers of mosquitoes, achieving an approximate four-fold increase in throughput over the standard microscopical readout if only infection intensity is the desired output^15^.

Our data from a large number of infections indicate that oocyst intensity is a simple linear function of luminescence intensity. This is in keeping with our previous work that showed a linear relationship between oocyst counts and luminescence intensity, using a different promoter to drive expression of the reporter gene^15^. Oocysts are an intermediate parasite life-stage which even when developing concurrently in a host mosquito may differ in size and sporozoite content^30^,^31^,^32^. Whereas microscopy produces discrete data, luminescence measurements provide continuous data that reflect actual numbers of developing sporozoites, which may be more directly relatable to mosquito infectivity, for which oocyst counts are an accepted proxy. An added advantage of the luminescent assay is that it is less subjective than microscopy based assessments of oocyst numbers. We cannot exclude the possibility that in heavily infected mosquitoes, with higher oocyst intensities than those reported here, nutrient and space competition may lead to lower *per capita* sporozoite production, resulting in a non-linear relationship between luminescence and oocyst intensity and lower luminescence based TRA estimates than would be determined from oocyst number alone. Though we advise caution in extrapolating actual oocyst number from luminescence values, we believe luminescence based TRA estimates may be more accurate than those made using oocyst data.

In the direct mode of the SMFA the compound remains present in the bloodmeal, and there is a remote possibility that it interferes with activity of the luciferase reporter rather than with oocyst development. We have been unable to investigate the potential effect of compounds interacting with *hsp70* expression, as has been observed in other eukaryotes^33^, or the possibility that compounds may directly inhibit luciferase protein after its production^34^. We consider both events highly unlikely to affect the luciferase readout in the current SMFA format, as compounds are at latest added to the mosquito bloodmeal 8 days before luminescence measurements. Furthermore, analyses of a subset of the MMV malaria box compounds in a luciferase inhibition assay did not reveal inhibitory activity at the concentrations that reduce oocyst development^22^. The possibility that some compounds may effect mosquito metabolism, and by proxy parasite developmental success, can also not be excluded. Readouts based on oocyst number may be insensitive to such subtle effects, while the more quantitative luminescence readout may not be. Additional experiments will be required to elucidate the interaction of mosquito and parasite fitness in relation to compound exposure.

Apart from oocyst intensity, oocyst prevalence (the proportion of mosquitoes that develop an oocyst-stage infection), is an important parameter determining malaria transmission. Whereas inter-experimental variation in control mosquito oocyst burden has no effect on TRA expressed as a reduction in oocyst intensity, it heavily influences the outcome based on a reduction in oocyst prevalence^35^. Since our experiments comprised a large set of experimental infections that each showed a distinct distribution of oocyst densities we have, therefore, limited our analyses to a comparison of compound effects on oocyst intensity, as this is the only parameter that is directly comparable between experiments. Nevertheless, a complete assessment of the transmission blocking potential of an antimalarial would require a description of the effects on infection prevalence, preferable at infection intensities that mimic those observed in naturally infected mosquitoes^36^. An experimental and computational strategy to achieve such an assessment has been implemented in our laboratory and will be described elsewhere.

Compounds reducing malaria parasite transmission to mosquitoes may kill gametocytes directly (gametocytocidal activity), irreversibly impair the parasites ability to develop once ingested by the mosquito, or else inhibit gametocyte developmental viability only while drug pressure is maintained in the mosquito environment^12^. Because mature gametocytes persist at low densities for several weeks after ACT treatment^6^, and because submicroscopic gametocyte densities may be highly transmissible^37^,^38^, efforts to develop transmission-reducing compounds are focused on identifying activity against mature gametocytes^39^. *In vitro* assays based on markers of metabolic activity (pLDH^13^,^19^, Alamar Blue reduction^20^) or reporter genes^21^,^22^,^40^ can be used to assess the effects of gametocytocidal compounds but may overlook compounds that manifest their action downstream of the gametocyte. Gametocyte infectivity may be assessed in greater detail with gamete formation assays, which can include gametocyte washing steps to discriminate between reversible and irreversible compound activity^12^,^13^,^23^. *In vitro* ookinete formation assays can also be used to identify compounds whose presence in the mosquito blood meal inhibits parasite fertilisation^41^, but these assays cannot exclude the possibility of compounds reducing ookinete viability or oocyst formation. Assays measuring the viability or development of gametocytes without mosquito feeding are comparatively inexpensive and highly scalable, so will continue to be required as a prioritization step in the screening pipeline for transmission blocking drugs. The data presented here indicate, however, that increased throughput of *in vitro* gametocyte assays goes at the cost of a considerable number of false negatives. Importantly, as no *in vitro* assay or combination of assays is able to fully interrogate the effect of compounds on all parasite developmental stages exposed to test compounds, our results underline that the SMFA remains the gold standard assessment of gametocyte infectivity. Our indirect screen of a varied set of 47 antimalarials identified compounds preventing mosquito infection by mechanisms targeting any stage of the parasites sexual development or early sporogony. Assays based on parasite metabolic processes (pLDH) and on the expression of female gamete upregulated protein Pfs25 cumulatively failed to identify seven compounds which, incubated with cultured gametocytes (NF54-HGL) in the same concentration, caused marked reductions in oocyst stage productivity as assessed by the luminescent SMFA. Though we anticipated identifying compounds with additional activity downstream of gamete formation in the SMFA, the results of the direct assay indicate that the pLDH and gamete formation assays failed to identify compounds with effects restricted to the gametocyte; three of the seven false negatives had no activity on mosquito stage parasites in the direct SMFA format. The effect of the remaining four compounds may rely on a sporontocidal rather than a gametocytocidal activity. In keeping with previous reports of their sporontocidal activity (reviewed in Butcher^42^), pyrimethamine and atovaquone blocked oocyst development but were not active in the pLDH and gamete formation assays. Atovaquone has demonstrated a long-lasting transmission-blocking effect in *ex vivo* studies with sera from drug-treated volunteers^43^, but the effect on transmission in malaria endemic settings has not been described in detail. The sporontocidal effect of pyrimethamine most likely represents a class effect of dihydrofolate reductase (DHFR) inhibitors, as it was also observed for P218 and chlorproguanil. Proguanil was understandably not active in the SMFA as only its active metabolite cycloguanil binds to DHFR^44^.

Analyses of eighteen compounds from the MMV malaria box revealed sixteen compounds with functional TRA that were not necessarily identified by individual gametocyte based assay formats^12^,^24^,^25^,^26^. These 18 compounds were flagged as potential transmission blockers by a joint effort coordinated by the Bill and Melinda Gates Foundation and the Medicines for Malaria Venture under the ‘Gametocyte Assays for *Plasmodium falciparum’* program. Under this program, five consortia consisting of two to five malaria laboratories each screened the MMV malaria box in a variety of gametocyte-based assays. All consortia proposed a shortlist of active molecules for further evaluation in the SMFA. The final selection of 18 molecules, governed by MMV, was then based on potency, diversity in chemical scaffolds and presumed tractability. As these compounds were expected to act at the level of the gametocyte, we applied a wash-out format to restrict the analysis to the sexual blood stage of the parasite when testing these compounds in the luminescent SMFA. For the 18 compounds subjected to this more rigorous investigation, 16 were able to reduce onward oocyst development in the mosquito with potencies ranging from 8.6 to 0.04 μM. The luminescent SMFA did not reveal TRA for MMV020492, which is in line with its lack of activity in published gametocyte assays. Equally inactive in the SMFA, MMV020492 showed a lack of activity in most published gametocyte assays, with the exception of a luciferase assay that indicated an IC50 of 62 nM. Overall, from a qualitative perspective the luciferase assay showed the best agreement with the SMFA, as it identified 14 out of the 16 compounds with functional TRA. Assays that interrogate the ability of a gametocyte to form a male or female gamete did not identify MMV000442 as a potential transmission reducing compound^12^, whereas it showed an IC50 of 590 nM in the SMFA. This indicates that gametocyte infectivity is defined by a greater set of functions than described by its sole ability to form a gamete. Quantitatively, neither of the published gametocyte assays correlated particularly well with the SMFA, as IC50 values could vary up to 180-fold (*e.g.* MMV007116 SMFA vs gametocyte exflagellation). In addition, the rank order of compounds appeared to be different for all assays. This implies that *in vitro* gametocyte assays provide limited guidance for studies aimed at an understanding of dose in relationship to efficacy. Although mouse models have been proposed to determine the efficacious dose for blockage of malaria transmission, these are restricted to *P. berghei* parasites and have limited value for predicting efficacy on human malaria. In addition, *P. berghei* infection intensities are much higher than *P. falciparum* infection intensities, which leads to an underestimation of TRA when estimated from differences in infection prevalence^35^,^45^.

**Figure 5:**
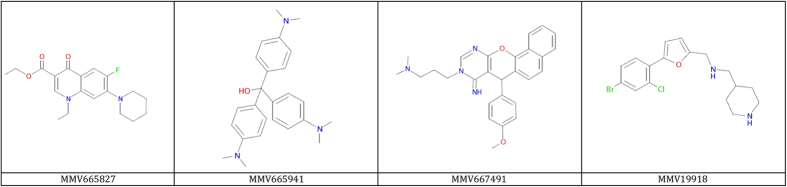
Potent transmission-blocking molecules. The depicted compounds showed an IC50 < 100 nM in the luminescent SMFA.

Our studies identified four compounds from the malaria box with IC50 values below 100 nM in the luminescent SMFA (Fig. 5). Compounds MMV665827, MMV665941, and MMV667491 lack structural properties that meet Lipinski’s rule of five that predicts oral absorption of a candidate drug^46^. Therefore, these compounds are considered tool compounds rather than drug-like molecules^47^. Nevertheless, these compounds may have value in the identification of mechanisms underlying transmission-blocking activity, which remain poorly understood. Importantly, these molecules have activity against asexual blood stage parasites, which opens the possibility of selecting drug resistant mutants followed by whole genome sequencing. This strategy has been successfully employed in the identification of PI4K, and eEF2 and confirmation of DHODH as bonafide drug targets^48^,^49^,^50^. From the group of compounds with potent transmission blocking activity, MMV19918 appears to qualify as a drug-like molecule and may, besides serving as a tool in unravelling mechanism of action, act as a starting point for further optimization. This compound appeared 10-fold more active in the SMFA as in assays interrogating activity against the asexual blood stages. Such a transmission-selective profile was also observed for MMV665941 and MMV667491. To the best of our knowledge these are the first molecules described to date that show selectivity towards the transmission stages of the parasite. Recently, the malaria drug development pipeline was enriched with molecules like OZ439, DSM265 and DDD107498, which unlike the majority of marketed antimalarials have activity against the transmission stages of the parasite^51^. However, for all these molecules this activity is at par or less potent than the activity observed against asexual blood stage parasites^13^,^49^,^50^,^52^. The observation of a transmission-selective profile in the study presented here suggests a novel mechanism of action outside the range of targets described to date. In view of the need for molecules that address a target candidate profile for prevention of transmission^53^, these mechanisms and the molecules identified here warrant further investigation.

## Conclusion

The results of the current study demonstrate the sensitivity and flexibility of the SMFA for determining the effect of small molecules on *P. falciparum* gametocyte transmission to mosquitoes, while validating a method that increases the assays scalability and ease of use. Comparison to gametocyte inhibition assays indicates that parasite development in the mosquito may be required to ensure assay sensitivity to transmission-blocking small molecules. Of the 18 compounds selected in this analysis based on the results of previous gametocyte assays, our data indicate that 3 compounds have greater activity on transmission stage parasites than asexual stage parasites. These molecules may be of particular interest for further drug discovery and elucidation of novel drug targets.

## Methods

### Parasites and culture

Asexual blood stages of all *P. falciparum* parasite lines were cultured in RPMI 1640 medium supplemented with 367 μM hypoxanthine, 25 mM HEPES, 25 mM sodium bicarbonate and 10% human type A serum, in a semi-automated system under standard conditions and induction of gametocyte production was performed as previously described^54^,^55^,^56^.

### Generation of NF54-HGL-hDHFR and NF54-HGL parasite lines

The GFP::LUC fusion protein under control of the *hsp*70 promoter was stably integrated into the genome of asexual stage NF54 (WT) parasites at the *pfs47* locus (PF13_0248) by standard double crossover recombination and positive/negative selection^57^,^58^,^59^. The targeting plasmid was based on the gene deletion construct pHHT-FRT-Pf5236 described previously^60^. The *p52* 3′ target region in pHHT-FRT-Pf5236 was replaced by the 3′ *pfs47* target region amplified by PCR using primers MWV257 and MWV259 (primer sequences in supplemental Table S1) and introduced in the plasmid by *MluI/SacII* restriction digestion and ligation. The 5′ *p52* region was replaced with the 5′*pfs47* target region amplified by PCR using primers MWV262 and MWV267 and introduced in the plasmid by *XmaI/NarI* restriction digestion and ligation. A reporter gene cassette was constructed by subcloning a GFP::LUC fragment from plasmid PL1063^61^ in pCR2.1-BLUNTII-TOPO after *AflII/XbaI* restriction digestion. An AgeI restriction site was introduced immediately upstream of the start codon by introducing a PCR fragment amplified with primers MWV252 and MWV253 through *KpnI/HpaI* restriction digestion and ligation. The promoter region of the *hsp70* gene (PF3D7_0818900) was placed upstream of GFP through *sacII/AgeI* restriction digestion and ligation after amplification with primers MWV198 and MWV200 and subcloning in pEGFP-N1 (Clontech laboratories, Mountain view, CA) through *BamHI/SacII* digestion and ligation. The 3′ transcription termination region from the *hsp86* gene (PF3D7_0708400) was introduced following PCR amplification using primers MWV268 and MWV271 and *XbaI/ApaI* restriction digestion and ligation. Finally the reporter cassette was introduced in the gene targeting plasmid by *KpnI/NgoMIV* restriction digestion and ligation yielding plasmid pMV163.

Pfs47 target regions, 5′hsp70 and 3′hsp86 fragments were amplified by PCR amplification (Phusion, Finnzymes) from genomic *P. falciparum* DNA (NF54 strain) and all PCR fragments were sequenced after TOPO TA (Invitrogen) sub-cloning.

Transfection of wild type NF54 asexual parasites with DNA construct pMV163 was performed as described previously^59^ using a Electro Cell Manipulator 600 (BTX, Holliston, USA) Double crossover mutants were selected by positive and negative selection as described^57^, yielding parasite line NF54-HGL-hDHFR. Cloning of transgenic parasites was performed by limiting dilution in 96-well plates as described^62^. Parasites in positive wells were transferred to the semi-automated culture system and cultured for further phenotype and genotype analyses.

Removal of the hdhfr selection marker was next performed by transfection of the mutants with construct pMV-FLPe and selection on blasticidin^60^,^63^, resulting in parasite line NF54-HGL. Integrity of the transgenes was analysed by PCR using primers MWV300 and MWV301 and *XcmI*/*NcoI* restriction digestion.

To confirm functional FRT-mediated excision of the hDHFR selection marker, sensitivity to anti-folates and a number of non-folate reference compounds were performed on NF54 (WT), NF54-HGL-hDHFR, and NF54-HGL using a modified pLDH assay. Briefly, non-synchronised asexual parasites (~50% ring stages, 25% trophozoites and 25% schizonts) were seeded at a density of 0.5% parasitaemia in 3% haematocrit in a black 96-well plate in 50 μl of culture medium, with 50 μl of diluted test compound. Following a 72 hour incubation at 37 °C, 98% humidity, 93% N2, 4% CO2 and 3% O2, the increase in parasitaemia was assessed using the modified pLDH assay as described previously^13^. Assays were performed in triplicate (three serial dilutions for each compound) and all plates included triplicate controls to assess minimum (MIN control, 1 μM DHA) and maximum (MAX control, 0.1% DMSO) signal. To compare the three different strains data were normalized and expressed as the percentage reduction in replication relative to the blocking and non-blocking controls.

### GFP fluorescence analysis

The midguts of mosquitoes fed previously with NF54-HGL gametocyte culture were mounted on glass slides in a droplet of PBS, and GFP expression was visualized and photographed on a Leica fluorescence microscope with digital camera.

### Standard Membrane Feeding Assays

In the ‘indirect’ mode of the SMFA, gametocytes were pre-incubated with test and control compounds prior to mosquito feeding. To this end, compounds were dissolved in DMSO to achieve a stock solution of 10 mM that was serially diluted in DMSO to achieve concentrations 1000-fold above the final test concentration. Subsequently, 10 μl of diluted compound in DMSO was added to 990 μl pre-warmed RPMI 1640 medium supplemented with 367 μM hypoxanthine, 25 mM HEPES and 25 mM NaHCO_3_ (‘incomplete medium’). 40 μl of this intermediate dilution was added to 360 μl of parasite culture and incubated for 24 hrs at 37 °C in Eppendorf tubes, resulting in 0.1% final DMSO concentration. Hereafter, 300 μl of the gametocyte culture/compound mix was added to 180 μl of packed red blood cells and centrifuged for 20 seconds at 10,000 × *g*. After carefully aspirating the supernatant, 200 μl of human serum type A was added to the pellet. Finally, 300 μl of this mix was immediately injected into an individual membrane covered minifeeder, where 50 female *A. stephensi* mosquitoes were allowed to feed for 10 minutes. In the ‘wash-out’ mode of the SMFA, compounds were washed out following preincubation by adding 14 ml incomplete medium supplemented with 10% human type A serum (‘complete medium’) to the gametocyte suspension, followed by centrifugation (2000 × *g*) for 7 minutes. The supernatant was removed and the gametocytes were washed again with 14 ml of complete medium. The pellet was resuspended in 300 μl of complete medium and combined with 180 μl packed red blood cells. All media used for washing were prewarmed to 37 °C and the subsequent preparation of the blood meal was as described above. For the ‘direct’ mode of the SMFA, 300 μl parasite culture was added to 180 μl red blood cells, centrifuged for 20 seconds at 10,000 g and the supernatant was carefully aspirated. Meanwhile, 10 μl of compound diluted in incomplete medium was added to 490 μl human serum type A (0.1% DMSO final). 200 μl of this human serum/compound mix was added to the gametocyte culture pellet, which was subsequently used for the SMFA as described above. For dose response analyses, compounds were diluted in DMSO with subsequent dilution in culture medium to a final DMSO concentration of 0.1%. Controls included vehicle (0.1% DMSO) and DHA at its approximate IC90 (10 μM). For all assay types, unfed and partially fed mosquitoes were removed from the cage after feeding, and fed mosquitoes were maintained at 26 °C, 80% humidity. Mosquitoes were either dissected and examined for midgut oocysts on day 6–8 PI as described^64^,^65^, or processed in the luciferase activity assay on day 8 PI.

### Mosquito processing and luciferase assay

To enhance flexibility, and to ensure that individual mosquitoes were safely and efficiently transferred from feeding cages to 96-well plates for homogenisation, mosquitoes were immobilised at day 8 PI and stored at −20 °C in sealed 15 ml falcon tubes for at least 24 hours and up to 2 weeks, before being processed in the luciferase assay. Upon removal from storage at −20 °C mosquitoes were thawed and individually added to wells in shallow 96-well plates containing 60 μl PBS with Complete protease inhibitor cocktail according to the instructions of the manufacturer (Roche, Switzerland), and 0.2 gram of 1.0 mm diameter zirconia beads (Lab Services BV). Plates were next placed in a Mini-Beadbeater-96 (Biospec, Bartlesville, OK) and rocked for 2*15 seconds, with 1–2 minutes on ice between rounds of homogenisation. Based on visual inspection, this procedure achieved a homogenate consistency in line with that achievable using a hand-held pestle rotator as described previously^15^. For the individual mosquitoes 45 μl of homogenised material was transferred to wells in a Krystal 2000 black & white 96-well plate (Porvair, United Kingdom). Next 45 μl of Bright-Glo luciferase assay substrate (Promega, Madison, WI) was added to each well using a Cybi Selma dispensing station (Cybio, Germany) and the plate was incubated for 3 minutes at room temperature. Luciferase activity was measured using a Synergy 2 multi-purpose plate reader (Biotek, Winooski, VT). Background was determined by measuring uninfected mosquitoes that had undergone the same treatment.

### pLDH and Pfs25 assays

Assays were performed as described previously^13^.

### Data analysis

Statistical analysis was conducted using R (Foundation for Statistical Computing, Vienna, Austria), STATA 12 (StataCorp., TX, USA) and GraphPad Prism 5.0 (GraphPad Software Inc., CA, USA).

All comparisons of microscopy and luciferase outcomes were conducted with separate groups of mosquitoes for each measure sampled from the same feeds. Comparisons between oocyst and luminescence based measures of mosquito infection prevalence were made with Chi-squared tests with Bonferonni correction. Associations between oocyst and luminescence intensity were quantified with linear regression. 95% confidence intervals around mean luminescence and oocyst intensity data was calculated by bootstrapping (1000 repetitions). Luminescence and microscopical TRA estimates were made using generalised linear mixed models (GLMMs)^35^,^66^. For assessment of infection prevalence, the cut-off for RLU based infection positivity was determined arbitrarily as the mean luminescence intensity of all uninfected controls plus five standard deviations (12.7 RLU), as described previously^15^. For gametocyte viability and gametogenesis assays, data were expressed as the percentage effect relative to the MIN and MAX controls^13^. For SMFAs, data were expressed relative to the negative (vehicle) controls for oocyst density and oocyst prevalence (i.e. the proportion of infected mosquitoes). IC50 values were calculated by applying a four-parameter logistic regression model using a Maximum Likelihood Estimation algorithm to find the best fit.

## Additional Information

**How to cite this article**: Vos, M. W. *et al.* A semi-automated luminescence based standard membrane feeding assay identifies novel small molecules that inhibit transmission of malaria parasites by mosquitoes. *Sci. Rep.* **5**, 18704; doi: 10.1038/srep18704 (2015).

## Supplementary Material

Supplementary Information

## Figures and Tables

**Table 1:** Compound activity as determined in the luminescent SMFA compared to activities reported by published gametocyte viability assays and asexual replication assays.

Compound	washout SMFA		direct SMFA	published gametocyte assay data					asexual blood stage^†^
	IC50 μM (95% CI)	slope (95% CI)	% inh. 10 μM	exflagellation^12^ (imaging) IC50 μM (% inh. 1 μM)	Pfs25^12^ IC50 μM (% inh. 1 μM)	alamarBlue^24^ IC50 μM (% inh. 5 μM)	imaging^25^ IC50 μM (% inh. 5 μM)	exflagellation^26^ (SYBR green) IC50 μM (% inh. 10 μM)	IC50 (μM)
MMV000248	1.64 (1.26 − 2.13)	-2.44 (-3.07 − -1.81)	48.76^*^	NA (-66%)	NA (8%)	0.31	1.1	NA (95%)	0.72
MMV000442	0.59 (.35 − 1.01)	-1.18 (-1.58 − -0.78)	5.50	NA (-12%)	NA (6%)	NA (72%)	0.92 (55%)	NA (59%)	0.36
MMV000448	1.18 (1.07− 1.31)	-11.63 (-15.91 − -7.34)	97.93^*^	0.25	4.02	1	0.71	5.4	0.23
MMV007116	0.11 (.06 − .19)	-1.62 (-2.15 − -1.08)	99.22^*^	20.75	NA (10%)	NA (40%)	0.26	NA (67%)	0.35
MMV011438	8.58 (7.95 − 9.26)	-8.47 (-12.62 − -4.32)	-3.29	NA (-77%)	NA (-31%)	0.82	1.13	NA (69%)	0.32
MMV019266	1.29 (1.21 − 1.38)	-9.38 (-12.41 − -6.35)	7.07	NA (31%)	NA (-11%)	0.31	0.33	NA (81%)	0.62
MMV019881	1.17 (. −.)	-15.81 (. −.)	88.15^*^	NA (57%)	NA (-7%)	NA (76%)	NA (32%)	5.5	0.65
MMV019918	0.07 (.04 − .13)	-1.36 (-1.80 − -0.93)	88.55^*^	0.07	0.52	0.32	0.7	0.9	0.79
MMV020492	ND	ND	-16.63	NA (36%)	NA (-12%)	NA (70%)	NA (-5%)	NA (59%)	0.03
MMV396797	3.62 (.87 − 15.18)	-4.10 (-9.87 − 1.67)	-16.44	NA (-14%)	NA (-17%)	NA (90%)	0.01	8.8	0.48
MMV665827	0.10 (.06 −.15)	-2.23 (-3.05 − -1.42)	98.25^*^	16.55	NA (-15%)	NA (41%)	0.34	NA (66%)	0.12
MMV665882	ND	ND	4.92	NA (-12%)	NA (-48%)	NA (92%)	0.07	NA (75%)	0.47
MMV665941	0.04 (. − .)	-10.29 (. − .)	99.84^*^	0.42	0.3	NA (86%)	0.32	1.8	0.26
MMV665971	3.22 (1.45 − 7.15)	-1.50 (-2.57 − -0.44)	-7.04	NA (–29%)	NA (–13%)	NA (73%)	0.23	NA (61%)	0.49
MMV665980	1.76 (1.57 − 1.96)	-11.52 (-25.29 − 2.24)	64.17^*^	8.07	NA (74%)	NA (27%)	NA (91%)	NA (76%)	0.21
MMV666021	1.25 (1.17 − 1.34)	-13.30 (-17.32 − -9.28)	94.02^*^	NA (30%)	NA (–20%)	NA (58%)	0.61	NA (68%)	0.09
MMV666125	1.40 (1.11 − 1.76)	-2.86 (-3.97 − -1.76)	-24.69	NA (–3%)	NA (–17%)	0.48	0.75	NA (74%)	0.38
MMV667491	0.06 (.03 − .11)	-0.61 (-0.73 − -0.50)	89.05^*^	0.09	0.18	NA (91%)	1.06	4.5	1.23

The table shows IC50 values where available (NA: not available) or the percentage inhibition at the highest concentration tested, as indicated in the header row. IC50 data for the exflagellation (imaging) and Pfs25 assays were generated in a washout format, whereas percentage inhibition values represent data from a continued exposure experiment^12^. For SMFA data, 95% confidence intervals are indicated between brackets where possible. For MMV019881, and MMV665941 the model did not converge. Asterisks (^*^) indicate statistically significant inhibition (p < 0.05) in the direct SMFA. ^†^ The last column shows activity against asexual blood stages of *P. falciparum* strain 3D7 as deposited at the ChEMBL repository under accession number CHEMBL2028071. ND: no dose response relationship was detected in the data.
