# Supplementary material for: A semi-automated luminescence based standard membrane feeding assay identifies novel small molecules that inhibit transmission of malaria parasites by mosquitoes
Source: Sci Rep. 2015 Dec 21;5:18704. doi: 10.1038/srep18704 (PMC4685452; doi:10.1038/srep18704)
Supplement: Supplementary Information [file srep18704-s1.pdf]

A semi-automated luminescence based standard membrane feeding assay identifies novel small molecules that inhibit transmission of malaria parasites by mosquitoes

Martijn W. Vos[1,†], Will J. R. Stone[2,†], Karin M. Koolen[1], Geert-Jan van Gemert[2], Ben van Schaijk[2,] Didier Leroy[3], Robert W. Sauerwein[1,2], Teun Bousema[2], Koen J. Dechering[1,*]

[1]TropIQ Health Sciences, PO Box 9101, 6500 HB Nijmegen, The Netherlands

[2]Department of Medical Microbiology, Radboud University Nijmegen Medical Center, 6500 HB Nijmegen, The Netherlands

[3]Medicines for Malaria Venture, 20 Route de Pré-Bois, 1215 Geneva 15, Switzerland

[†]contributed equally

[*]corresponding author. E-mail: k.dechering@tropiq.nl. Tel. +31-6-51483935

SUPPLEMENTARY INFORMATION

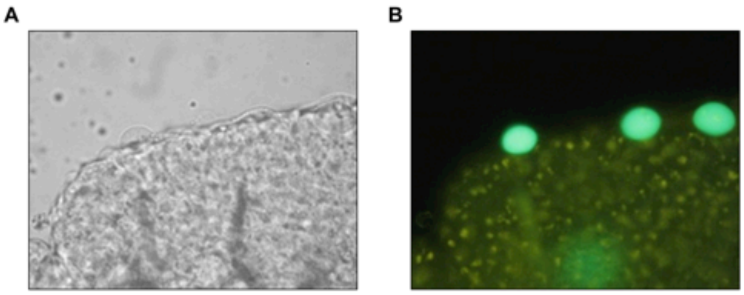

Figure S1. **A.** Brightfield micrograph of a section of mosquito midgut with 3 oocysts taken after mosquito dissection 6 days PI with NF54-HGL. **B.** Fluoresence micrograph of the same section of mosquito midgut with 3 oocysts taken after mosquito dissection 6 days PI with NF54-HGL.

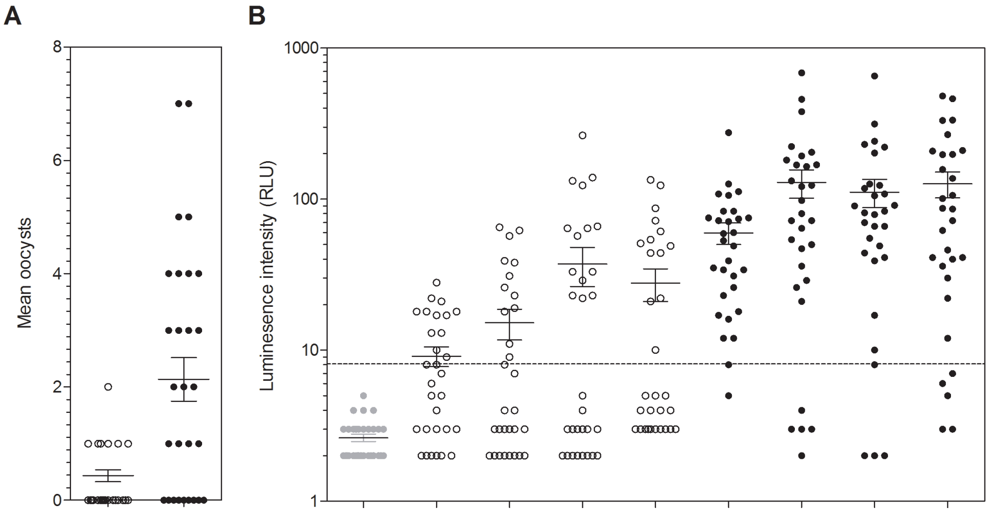

Feed	A	B
Day PI	8	8
Mean oocysts	0.43	2.13
Mean RLU	-	-
Prevalence	40%	70%

Neg	**A**	**A**	**A**	**A**	**B**	**B**	**B**	**B**
-	7	8	9	12	7	8	9	12
-	-	-	-	-	-	-	-	-
2.63	9.1	15.2	37.2	27.8	59.7	128.7	111.2	126.2
-	40%	40%	46.7%	43.3%	93.3%	83.3%	86.7%	83.3%

Figure S2. A. Oocyst intensity in individual mosquitoes from two groups of mosquitoes (*A* and *B*) infected with NF54-HGL, determined after dissection and standard midgut staining and microscopy at day 8 PI. **B.** Luminescence intensity in individual mosquitoes from the same two mosquito groups (*A* and *B*), assessed in separate mosquito samples removed from the primary mosquito storage cage at day 7, 8, 9 and 12 PI. The dashed line indicates the cut-off calculated statistically from the RLU values of all mosquitoes assayed in the current study (8.1 RLU). Prevalence estimates were based on this cut-off.

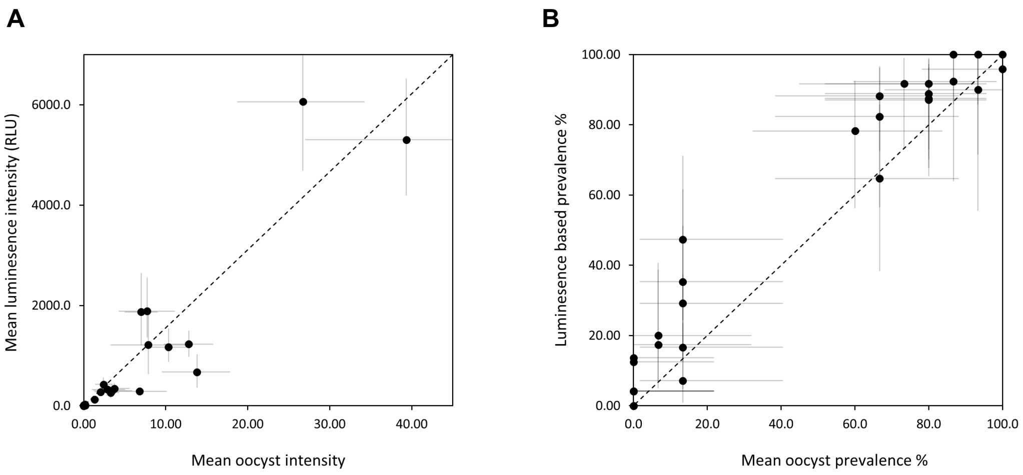

Figure S3. A. Relative oocyst and luminescence intensity among mosquitoes sampled from the same cages fed blood-meals without the addition of a test compound (16 feeds, 480 mosquitoes) or with the addition of DHA (16 feeds, 711 mosquitoes). **B.** Relative infection prevalence based on the observation of oocysts or calculated from luminescence intensity, among mosquitoes sampled from the same cages fed blood-meals without the addition of a test compound (16 feeds, 480 mosquitoes) or with the addition of DHA (16 feeds, 711 mosquitoes).

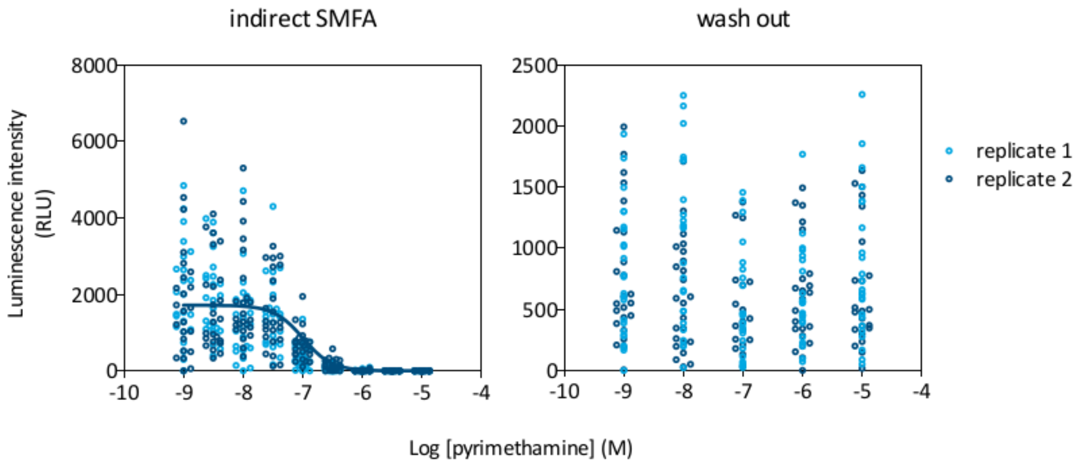

Figure S4. Efficacy of the washout protocol. The left panel shows the activity of pyrimethamine in the indirect SMFA as reported in Figure 2 of the main manuscript. The right panel shows the activity in the wash-out mode of the SMFA, where the compound was washed out prior to mosquito feeding.

**primer**	**sequence 5'-3'**
MWV 257	GTTAACGAAGTTCCTATACTATTTGAAGAATAGGAACTTCACGCGTGCCGGCTATGGTACCGTACCTACTAACTGTTTTGAATCTC
MWV 259	CCGCGGTTCATTCATATGCTAACATACATG
MWV 262	GGCGCCGATTGGTAGTTGTACTCATCTTG
MWV 267	CGAATTCGAAGTTCCTATTCTCTAGAAAGTATAGGAACTTCCCGGGCATAGTATCTACATAACTGAAGCAAG
MWV 252	GGTACCACTAGTCCCGCGGATCAACCGGTATGAGTAAAGGAGAAGAACTTTTCACTGG
MWV 253	TTGAAGTTAACTTTGATTCCATTCTTTTGTTTGTCTG
MWV 198	TATCCCGCGGGCTAGCCGCATAAATATCTGGTGAAATAC
MWV 200	TATCGGATCCCCTTTTGCACTAGCCAATTTTTC
MWV 268	AATGGGCCCGAATTCGCCGGCTACCTTATTTGATGAATTAACTACAC
MWV 271	TCTAGATTGTCGACAGGATTTATATAATATATTTATGTACTCACAATGGG
MWV 300 (P1)	TACATTCAAATAACTCAGAGGGTAAC
MWV 301 (P2)	GTTTGTGTATATTTACCTTACATTTATCTCC

Table S1. List of DNA primer sequences as described in methods.
